# Mechanosensory Stimulation Evokes Acute Concussion-Like Behavior by Activating GIRKs Coupled to Muscarinic Receptors in a Simple Vertebrate

**DOI:** 10.1523/ENEURO.0073-17.2017

**Published:** 2017-04-27

**Authors:** Wen-Chang Li, Xiao-Yue Zhu, Emma Ritson

**Affiliations:** University of St Andrews, St Andrews, Fife KY16 9JP, Scotland

**Keywords:** brainstem, concussion, GIRK, mechanosensory, muscarinic, swimming

## Abstract

Most vertebrates show concussion responses when their heads are hit suddenly by heavy objects. Previous studies have focused on the direct physical injuries to the neural tissue caused by the concussive blow. We study a similar behavior in a simple vertebrate, the *Xenopus laevis* tadpole. We find that concussion-like behavior can be reliably induced by the mechanosensory stimulation of the head skin without direct physical impacts on the brain. Head skin stimulation activates a cholinergic pathway which then opens G protein-coupled inward-rectifying potassium channels (GIRKs) via postsynaptic M_2_ muscarinic receptors to inhibit brainstem neurons critical for the initiation and maintenance of swimming for up to minutes and can explain many features commonly observed immediately after concussion. We propose that some acute symptoms of concussion in vertebrates can be explained by the opening of GIRKs following mechanosensory stimulation to the head.

## Visual Abstract

**Figure:**
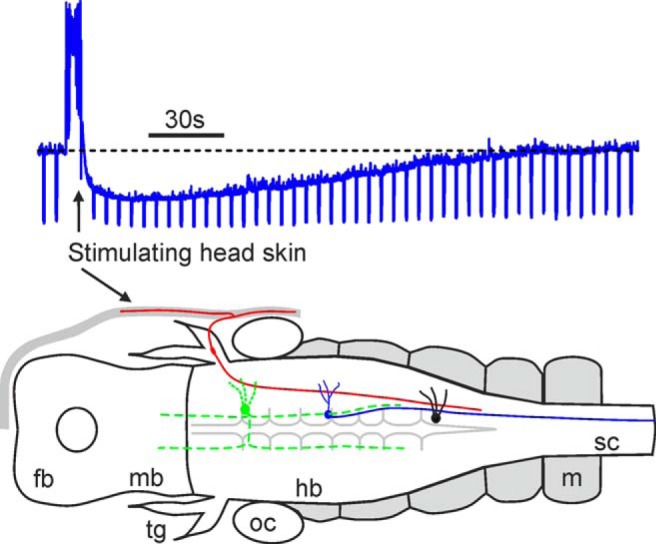

## Significance Statement

Most vertebrates have concussion responses when their heads are hit suddenly by heavy objects, rendering the animals momentarily motionless and often unconscious. We study a similar behavior in a simple vertebrate, *Xenopus laevis* tadpoles, and find that concussion-like behavior in these tadpoles can be induced reliably by mechanosensory stimulation of the head skin. The head skin stimulation then activates some cholinergic neurons in the brainstem to inhibit the tadpole motor circuit. These results provide a potential explanation why concussion in vertebrates often recovers spontaneously without sustaining clear physical injury to the brain and some acute symptoms of concussion can be a neurophysiological response to specific sensory stimulation.

## Introduction

When birds fly into glass windows or deer run into tree trunks, their movement often stops abruptly. These animals normally remain motionless and unconscious (knockout, KO) momentarily but recover spontaneously minutes later. Similar KO responses are also common in contact sports like boxing and American football. KO meets the criteria for concussion or mild traumatic brain injury (Hutchison et al., 2014). Mild cases of KO can undergo rapid recovery of neural functions where motor performance, learning, and memory are not affected (Parkinson et al., 1978). One intriguing feature of concussion is the temporary loss of motor or other brain functions without clear damage or injury to the brain (Trotter, 1924; Denny-Brown and Russell, 1941; Shetter and Demakas, 1979; Shaw, 2002; McCrory et al., 2009).

Although there is no consensus on what cellular mechanisms mediate the concussion responses, it is widely accepted that the pathology of concussion lies in the direct biomechanical damage to the brain inflicted by the concussive blow. Using different animal models, mostly mammals or primates under anesthesia, at least five hypotheses have been proposed to explain how concussion is caused by the sudden acceleration or deceleration of the brain (Shaw, 2002; Blennow et al., 2012; Zhang et al., 2014; Bolouri and Zetterberg, 2015). The vascular hypothesis has attributed the loss of consciousness to a brief episode of cerebral ischemia (Scott, 1940), but this has now been widely dismissed (Trotter, 1924; Denny-Brown and Russell, 1941; Nilsson and Pontén, 1977). Three other hypotheses have focused on the direct biomechanical insults to the brainstem, where some critical groups of neurons controlling arousal/sleep are located: reticular hypothesis (Foltz and Schmidt, 1956; Povlishock et al., 1983; Povlishock, 1986), centripetal hypothesis (Ommaya et al., 1964; Ommaya and Gennarelli, 1974; Adams et al., 1977), and pontine cholinergic system hypothesis (Hayes et al., 1984; Katayama et al., 1984). The convulsive hypothesis (Walker et al., 1944) has received the most popular support at the moment, and this proposes that mechanically elicited neuronal excitation explains the initial convulsive neuronal activity after the concussive blow; the following neuronal “exhaustion” accounts for the subsequent salient period of paralysis, muscle relaxation, behavioral stupor, and depressed cortical rhythms (Giza and Hovda, 2001; Shaw, 2002).

*Xenopus laevis* tadpoles at two days old (just hatched) display behavior similar to KOs when they swim into solid objects, i.e., their swimming stops abruptly and their motor responses are subdued afterward for many seconds. At this early developmental stage, the tadpole nervous system only has ∼4000 neurons. The neuronal circuits underlying swimming and most of the sensory responses have been defined (Roberts et al., 2010). Among the categorized neurons, the excitatory descending neurons (dINs) have been shown to play a critical role in driving the tadpole swimming rhythms (Li et al., 2006; Soffe et al., 2009). Due to extensive electrical coupling among dINs (Li et al., 2009), injecting depolarizing currents into a single dIN can occasionally initiate swimming and hyperpolarising currents into a single dIN can terminate on-going swimming (Moult et al., 2013). Other types of neurons rhythmically active during swimming (non-dINs) have similar intrinsic properties and their activity is driven by dIN EPSPs (Roberts et al., 2010). In this study, we have devised protocols to simulate tadpole KO behavior to investigate its underlying mechanisms and discuss its relevance to concussion in other animal models.

## Materials and Methods

Details of methods have been given previously (Li and Moult, 2012). In brief, pairs of adult male and female *X. laevis* were injected with human chorionic gonadotropin to induce mating, and embryos were maintained at different temperatures to stagger their development rates. All experimental procedures were approved by local Animal Welfare Ethics committee and comply with United Kingdom Home Office regulations. Tadpoles at two days old (stage 37/38, sex unidentifiable) were anaesthetised with 0.1% MS-222 (3-aminobenzoic acid ester, Sigma), then pinned onto a rubber stage in a dissection bath. The dorsal fin was cut to allow access of α-bungarotoxin (10 µM), which specifically binds with nicotinic receptors at the tadpole neuromuscular junctions (Li et al., 2004b; Li et al., 2014). After immobilization with α-bungarotoxin, the tadpole was re-pinned in the dissection bath and further cuts were made to remove ependymal cells from the inside of the hindbrain to expose neuronal cell bodies. Much of the belly yolk was removed to allow later visualisation of the exposed neurons on an upright Nikon E600FN2 microscope. The tadpole then was transferred to a small recording chamber with a rotatable Sylgard stage. Saline (127 mM NaCl, 3 mM KCl, 2 mM CaCl_2_, 2.4 mM NaHCO_3_, 1 mM MgCl_2_, and 10 mM HEPES, adjusted with 5 M NaOH to pH 7.4) in the chamber was circulated at ∼2 ml/min. Extracellular recordings of motor nerve activity were made from an intermyotomal cleft using a glass suction electrode to monitor tadpole motor outputs and KO responses. The dimming of a white LED light positioned close to the tadpole head was controlled directly by a Power1401 digitizer (Cambridge Electronic Design) to evoke fictive swimming. A stimulating glass suction electrode was placed on the head skin to induce the KO response.

Natural tadpole swimming behavior was observed in a 5-cm dish with 1-mm grid paper underneath and videoed at 120 or 240 fps using a GoPro HERO4 silver camera. A current amplifier was used to drive a speaker, whose membrane was attached to a glass rod (end blob: 100–400 µm in diameter). Half a cycle of sinewave current was used to drive the glass blob to tap the tadpole forehead to simulate its physical head-on clashes with a solid surface. The speed of tap could be adjusted by changing the sinewave frequency to match natural swimming speed (∼36 mm/s). To exclude the involvement of the cement gland in KO responses, the tadpole skin was removed except the head region. The tadpole was then pinned down onto the edge of a Sylgard stage, avoiding direct contact between the remaining head skin and the Sylgard. Similar taps were also applied to the tadpole head skin in immobilized tadpoles while whole-cell recordings were made. Electrical skin stimulation was delivered by a DS3 stimulator (Digitimer) to induce the KO response. During head-on clashes with Petri dish walls, the tadpole head touches the wall every time its tail flapped to propel the animal forward. Therefore, we set a typical electrical skin stimulation protocol as five pulses with duration of 0.2 ms at 30 Hz (tadpole swimming frequency ranges from 10 to 30 Hz) to approximately simulate the natural head-on clashes. The protocol was modified slightly during individual experiments (1–30 current pulses at 20–40 Hz, capped at 320 µA), depending on the sensitivity of the chosen stimulation site, to explore the best combination of parameters in inducing KO responses/inhibition or to maintain a consistent KO output for physiological and pharmacological tests. For example, the number of stimuli was increased to 10 when we mapped the sensitivity of different skin areas for the induction of KO responses. The stimulus number was reduced to three but frequency was increased to 40 Hz to avoid evoking more than one skin impulse in experiments for testing the involvement of skin impulses in the KO induction.

Vision-guided whole-cell recordings were conducted under the Nikon E600FN2 microscope. Patch pipettes were filled with an intracellular solution containing 0.1% neurobiotin (100 mM K-gluconate, 2 mM MgCl_2_, 10 mM EGTA, 10 mM HEPES, 3 mM Na_2_ATP, and 0.5 mM NaGTP adjusted to pH 7.3 with KOH). A junctional potential of 14.7 mV in standard saline was not corrected in voltage-clamp recordings. to chelate intracellular Ca^2+^ to block the activation of Ca^2+^-dependent potassium channels, pipette solution contained 20 mM BAPTA-4K^+^, 20 mM K-gluconate, 2 mM MgCl_2_, 2 mM EGTA, 10 mM HEPES, 3 mM Na_2_ATP, and 0.5 mM NaGTP. Signals were recorded with a multiclamp 700B amplifier and acquired with the Signal 5 software through the Power1401 digitizer at a sampling rate of 10 kHz. Stimuli to the skin were controlled using Power1401 configured by Signal. Cellular input resistance (R_inp_) was estimated by injecting 500-ms step currents at 0.2 Hz throughout the recordings, the amplitude of which was adjusted individually during experiments. Membrane conductance was calculated as the reverse of R_inp_. For skin impulse recordings, microelectrodes filled with 3 M KAC with a DC resistance around 150 MΩ were used. In these cases, three stimulation pulses at 40 Hz were applied to induce KO responses to avoid evoking more than one skin impulse, which has a duration of ∼100 ms. Post-stimulation swimming lengths were compared with trials where one stimulus was used and a single skin impulse was generated. To avoid activating lateral line sensory neurons, the tadpole head skin was peeled back, exposing the otic capsules as well as the trigeminal and lateral line nerves. After removing the otic capsules, lateral line nerves on both sides and the trigeminal nerve on one side were severed. A suction electrode was then placed on the remaining trigeminal ganglion to directly activate the mechanosensory neurons. When the evaluation of KO responses was solely based on motor nerve recordings, control swimming episodes were alternated 5–10 times with ones where KO stimuli were applied a few seconds after the initiation of swimming. The KO pharmacology was assessed by using different blockers via either bath application, where KO responses were monitored in motor nerve recordings, or microperfusion, where membrane conductance was monitored in whole-cell recordings. Wash-off measurements were made around 25 min or longer after the drug application had stopped. For intracellular application of BAPTA and GRK2i, measurements of membrane conductance were taken >15 min after whole-cell recordings were established. Microperfusion of Ba^2+^ and methoctramine was achieved by applying gentle pressure to the solution in a pipette with a tip diameter of 10–20 µm close to the recorded neuron. Tertiapin-Q was obtained from Alomone labs and other chemical agents were either from Sigma or Tocris.

Neurons were mostly recorded within the right side hindbrain. They were routinely stained for neurobiotin after fixation, treatment with Triton X-100 and incubation with extravidin peroxidase conjugate (Sigma-Aldrich). The nervous system was then dissected out with the notochord and some ventral muscles, dehydrated, cleared in methyl benzoate and xylene, and mounted whole between two coverslips using Depex. Neurons were observed on a normal histology microscope and traced using a drawing tube. Neuronal classification was based on the cellular anatomy and activity pattern during swimming (Roberts et al., 2010). All anatomic measurements were compensated for shrinkage due to dehydration by multiplying by 1.28.

Offline analyses were made with Dataview (courtesy of Dr. William Heitler in the University of St Andrews) and Excel. All data were tested for normality. Median values are given for non-normal data and drawn as box plots. Normal values were given as mean ± SE. Statistics were conducted using SPSS 22 (IBM).

## Results

**Figure 1.**
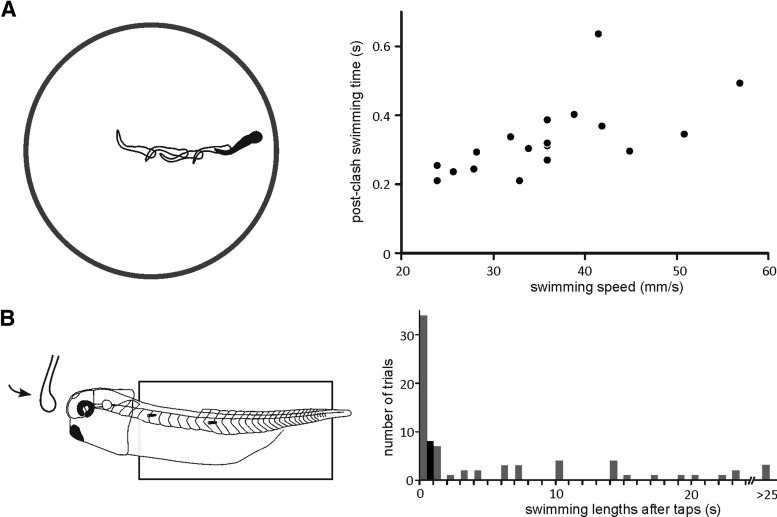
KO responses in free-swimming and restrained tadpoles. ***A***, KO responses videoed in a dish (left) with swimming speeds plotted against post-clash swimming lengths (right, 18 responses in six tadpoles, *p* < 0.01, two-tailed Pearson correlation). Post-clash swimming lasts for 0.33 ± 0.02 s (0.21–0.63 s). Tracing shows the tadpole swimming track. ***B***, Simulating head-on clashes in a physically restrained tadpole (left) and the distribution of post-tap swimming lengths. Gray represents single taps (half-cycle sinewave at 20 Hz) and black represents multiple taps (five sinewave cycles at 20 Hz).

We first measured how quickly tadpole swimming stopped after head-on clashes with a Petri dish wall using high speed videos (Video 1). Their swimming carried on for 0.33 ± 0.02 s (0.21-0.63 s) after the initial clash with an average of 5.4 ± 0.6 subsequent bumps (*n* = 6 tadpoles, 18 trials; Fig. 1*A*). We call this behavior KO for easier description. It was previously known that tadpole swimming could be stopped by the attachment of its cement gland to solid objects or the water surface (Roberts, 1980; Li et al., 2003). So we asked whether swimming was stopped by the activation of the cement gland in KO. An electronic tapping device consisting of a glass rod was used to simulate tadpole head-on clashes with a solid surface (Video 2), where the tap speed was controlled by the frequency of a half-cycle sinusoidal output. Single tap (half-cycle sinewave at 20 Hz) on the forehead of physically restrained tadpoles ended swimming after 1.33 s (range: 0.08–46.6 s, *n* = 21 tadpoles, 70 trials). Among them, swimming in 34/70 trials stopped <1 s after taps. Repetitive taps (five taps at 20 Hz) ended swimming after 0.15 ± 0.03 s (eight trials in three tadpoles; Fig. 1*B*), which was quicker than the single taps (*p* < 0.01, independent samples median test). The most effective way to activate the cement gland pathway is to directly prod the cement gland at low speed (Perrins et al., 2002; Li et al., 2003) or pull the mucus secreted from the gland (Lambert et al., 2004). The glass rod did not touch the cement gland or its mucus in the fast tap experiments, thus the cement gland pathway is not essential in KO responses. However, the tadpole normally lost its dorsal-side-up orientation after the initial clash with the Petri dish wall, allowing the potential attachment of cement gland mucus to the Petri dish. This may explain why the cessation of swimming was quicker and more reliable in head-on clashes than in fast tap experiments.

Video 1. Tadpole swimming is stopped by head-on clashes with a petri-dish wall. The grid lines are 5 mm apart. Swimming is started by dropping the tadpole in the petri dish using a plastic transfer pipette. 10.1523/ENEURO.0073-17.2017.video.1

Video 2. Tap the tadpole head with a glass rod stops ongoing swimming in a physically restrained tadpole. The tadpole is pinned through its notochord using three etched tungsten pins onto a sylgard stage. Swimming is started by dimming an LED light. 10.1523/ENEURO.0073-17.2017.video.2

**Figure 2.**
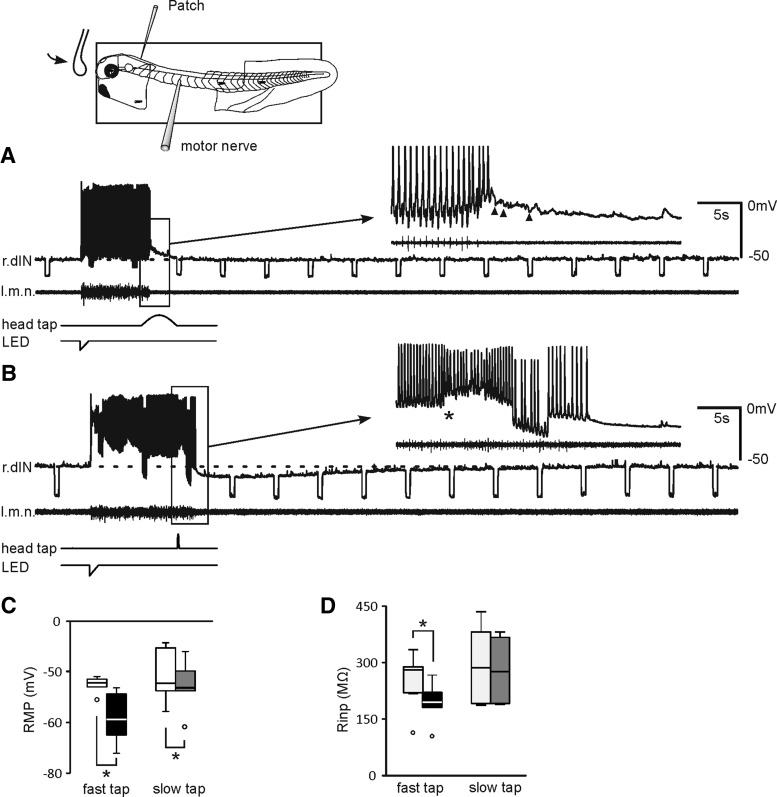
Responses of dINs to head taps. ***A***, A slow tap at 0.125 Hz ends swimming (motor nerve, m.n.) and evokes a mixture of EPSPs and IPSPs (arrow heads in inset) in a dIN on the right side of the hindbrain (r.dIN). ***B***, A fast tap at 2.5 Hz briefly excites another dIN in the right side hindbrain (*) then stops swimming with delayed, prolonged inhibition in the RMP and decreased R_inp_ (test steps: −100 pA). ***C***, ***D***, RMPs and R_inp_ in control (unfilled columns) and at the trough period of inhibition after taps. While slow taps at 0.125–0.25 Hz decrease RMPs in 8 dINs (from −52.2 ± 1.9 to −54.4 ± 1.9 mV, gray) without changing the R_inp_, fast taps at 2.5–10 Hz in 6 dINs decrease RMPs from −52.6 ± 0.7 to −59.1 ± 2 mV with a reduction in the R_inp_ by 29.6 ± 8.5% (black, *p < 0.05, paired *t* test or related sample Wilcoxon signed rank test). Dashed lines indicate RMP in ***A***, ***B***. Inset on the top shows experimental setup.

Whole-cell recordings were then conducted in immobilized tadpoles to identify the underlying KO mechanisms. First, single head-taps were used to identify which type of mechanosensory cells innervating the tadpole head skin were activated to initiate KO, rapid-transient detectors or slow movement detectors (Roberts, 1980). Among them, the activation of movement detectors by slow indentation to the head skin was previously shown to stop ongoing swimming (Roberts, 1980; Boothby and Roberts, 1992a). Unlike taps in restrained tadpoles, the tap speed (sinewave frequency) needed to be adjusted during each experiment to attain stable whole-cell recordings and effective stopping of fictive swimming. We found taps at different speeds/frequencies could stop swimming, but there were two types of neuronal responses. When fictive swimming was stopped by a slow tap driven by half a cycle of sinewave current at 0.25 or 0.125 Hz, neurons received both excitatory and inhibitory synaptic potentials (*n* = 15 neurons, 61 trials; Fig. 2*A*), similar to the prodding of tadpole cement gland (Li et al., 2003). This type of stopping was not investigated further in this study. Fictive swimming could also be stopped by a fast tap (2.5-10 Hz sinewave current), which tends to activate the transient detectors in the head skin (Roberts, 1980). In this case, neurons received a brief period of excitation followed by long-lasting inhibition (KO inhibition), with decreased R_inp_s (*n* = 6 dINs, 29 trials; Fig. 2*B–D*). Taps on the tadpole head at even higher speed caused immediate loss of whole-cell recordings, which were made <1 mm away. The average tadpole swimming speed is ∼36 mm/s (range, 24–57), roughly equivalent to the speed of a fast tap driven by 22.5 Hz sinewave currents. The tap experiments thus suggest KO responses are triggered by the activation of transient movement receptors in the head skin. To enable more stable recordings, we next electrically stimulated the head skin to evoke KO, normally a few seconds after swimming was initiated by dimming an LED.

**Figure 3.**
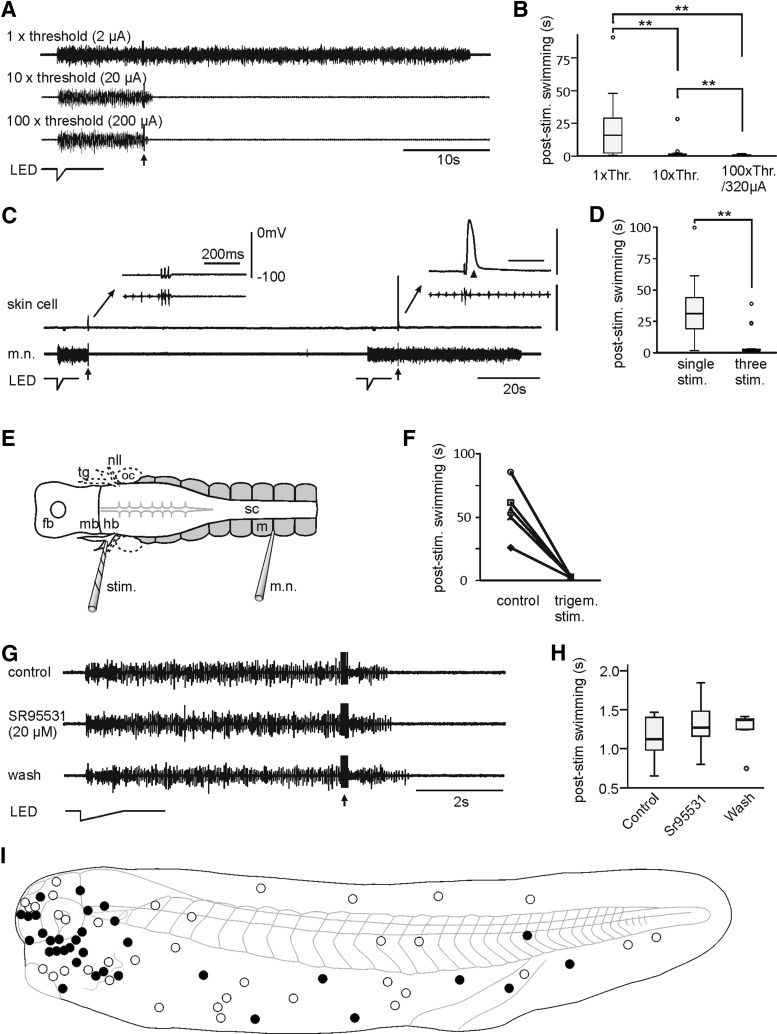
KO responses evoked by electrical skin stimulation. ***A***, Increasing skin stimulation (arrow, five pulses at 30 Hz) intensity shortens swimming in one tadpole. ***B***, Summary of poststimulation swimming lengths at different stimulation current intensities, capped below 320 µA, as shown in ***A***. Swimming thresholds at rest (Thr.) are 2–14 µA. ***C***, Three stimuli at 40 Hz (arrow, 55 µA) evokes a KO response in one tadpole without evoking any skin impulse, while a single stimulus (80 µA) evokes a skin impulse (arrowhead) without shortening swimming. Insets are stretched from time around stimulation. ***D***, Paired comparison of poststimulation swimming lengths as in ***C*** showing their dependence on the number of stimuli (*n* = 29 pairs from four tadpoles, *p* < 0.001, related samples Wilcoxon signed rank test). A single stimulus always evokes a skin impulse. ***E***, Experimental setup for the direct stimulation of the trigeminal ganglion (tg). A dorsal view of the CNS and some swimming myotomes are shown (fb, forebrain; mb, midbrain; hb, hindbrain; nll, lateral line nerve; stim., stimulation electrode; oc, otic capsule; sc, spinal cord; m, myotome). Dashed lines indicated severed nerves and oc. ***F***, KO responses evoked by stimulating the tg directly after removing nlls (*p* < 0.01 in each of five tadpoles, two-tailed independent sample *t* test). ***G***, Bath application of SR95531, a GABA_A_R antagonist, does not affect the KO response in a tadpole. KO stimuli (arrow) consist of five pulses at 30 Hz. ***H***, Summary of SR95531 experiments in six tadpoles (*p* > 0.05, related samples Friedman’s two-way ANOVA by ranks). ***I***, KO sites mapped with electrical skin stimuli (10 at 30 Hz). Each site has been applied KO stimuli for more than five times intercalated with trials without KO stimuli. Filled circles indicate KO stimuli shortened swimming (*p* < 0.05, paired *t* test or related samples Wilcoxon signed rank test) and empty circles indicate KO stimuli have no effect.

We used repetitive electrical skin stimulation (five 0.2-ms pulses at 30 Hz, KO stimulation) to simulate the multiple bumps occurring in the natural tadpole head-on clashes with solid objects (see above). At low current intensity, swimming after stimulation could normally last for a minute. KO-like shortening of swimming episodes was seen when larger stimulation currents were used (*n* = 13 sites in eight tadpoles, *p* < 0.0001, related samples Friedman’s two-way ANOVA by ranks; Fig. 3*A*,*B*). KO inhibition was always observed in experiments when there happened to be simultaneous whole-cell recordings of neurons in the rostral hindbrain. We therefore used swimming lengths after KO stimulation to assess KO effectiveness. In these experiments, KO stimulation was repeated at least five times to see whether it reliably shortened swimming. We next evaluated whether sensory systems other than the transient detectors were essential in the KO responses evoked by electrical skin stimulation. First, large stimulating currents can activate skin impulses, cardiac-like action potentials, and initiate swimming (Roberts, 1971). Skin impulses have duration of ∼100 ms; thus, they do not follow 40 Hz skin stimulation. We compared swimming evoked by a single skin stimulus with that evoked by three stimuli at 40 Hz while skin impulses were simultaneously monitored by recording directly from a skin cell using a sharp electrode. Skin impulses were evoked when the current intensity reached certain thresholds (5–100 µA), but KO responses were not dependent on them (Fig. 3*C*,*D*). Second, there are a couple of short rows of lateral line hair cells posterior to the tadpole eye. The electrical stimulation may indiscriminately activate the lateral line system. We peeled off the tadpole head skin and severed the lateral line nerves on both sides and the trigeminal nerve on one side (described in the methods). Stimulating the remaining trigeminal ganglion could still evoke KO responses (*n* = 5 tadpoles; Fig. 3*E*,*F*). Therefore, the lateral line system is not necessary in KO. Third, skin stimulation could activate slow-movement detectors, which stops swimming by activating mid-hindbrain reticulospinal GABAergic interneurons like the pressure sensors in the cement gland (Boothby and Roberts, 1992b). Bath application of 20 µM SR95531, a GABA_A_ receptor antagonist, failed to prevent KO responses (*n* = 6 tadpoles; Fig. 3*G*,*H*). This indicates that any activation of the skin slow-movement detectors and cement gland pathway is not essential in KO responses. Therefore, when skin stimulation currents are large, it is most likely that KO responses are mediated by the inhibition produced by activating transient movement detectors (Fig. 2*B*). When different areas of tadpole skin were systematically stimulated electrically, head regions showed the highest probability for KO induction (Fig. 3*I*; head 60%, trunk/tail 30%).

**Figure 4.**
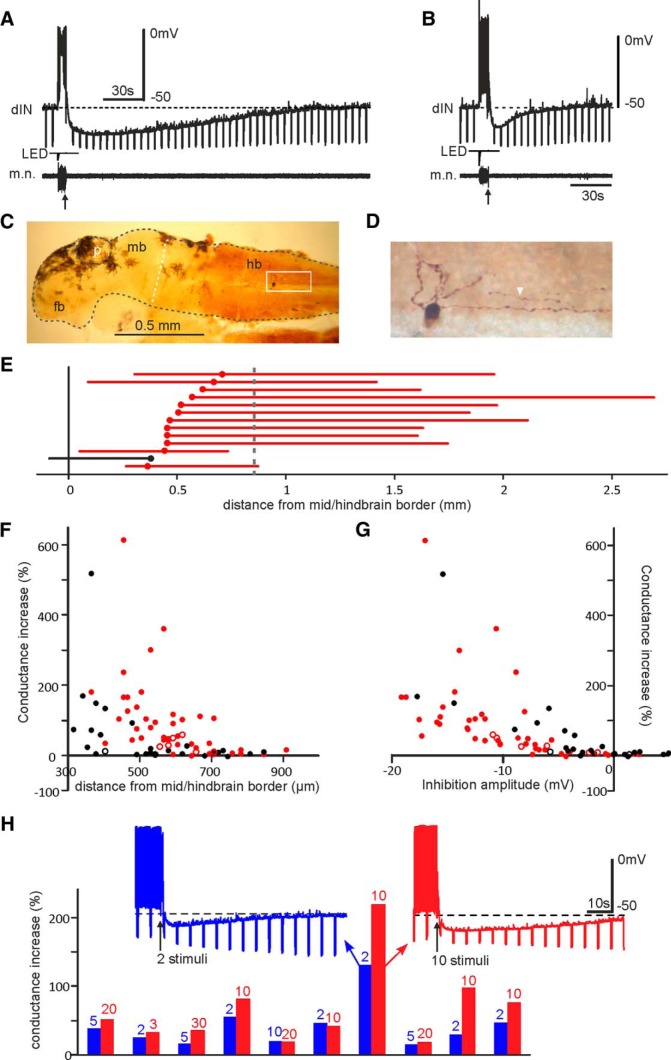
Properties of KO inhibition and their relation with the longitudinal location of neurons. ***A***, KO inhibition in a dIN 468 µm from mid/hindbrain border following electrical skin stimulation (five pulses at 30 Hz, arrow)**. *B***, KO inhibition after 10 electrical skin stimuli (25 Hz, arrow) in a dIN 670 µm from mid/hindbrain border. Dashed lines indicate RMP in ***A***, ***B***. ***C***, A dIN filled with neurobiotin in the hindbrain (hb, lateral view). Fb, forebrain; mb, midbrain; p, pineal eye. Black dashed lines outline the central nervous system. White dashed line marks the border between mb and hb (0 in ***E***). ***D***, Photo of the cell in ***C*** (white rectangle). Arrow head marks a short ascending axon. ***E***, The distribution of somata (circles) and axons (lines) of 13 neurons with >100% peak conductance increases during KO. Dashed vertical line marks the position of obex. ***F***, Longitudinal location of neurons plotted against their peak conductance increases during KO (48 dINs, 25 non-dINs, *p* < 0.001, two-tailed Spearman rank correlation coefficient 0.58). ***G***, KO inhibition amplitudes plotted against conductance increases (*p* < 0.0001, correlation coefficient 0.834). In ***E****–****G***, red symbols represent dINs; black symbols represent non-dINs. Hollow symbols are from tap experiments and solid ones from electrical skin stimulation. ***H***, Increasing the number of electrical skin stimuli (numerals above bars) while maintaining the stimulation strengths and frequencies induces larger rises in the membrane conductance in 10 dINs. The recordings from one dIN are shown in insets.

We next analyzed the properties of KO inhibition in whole-cell recordings. The inhibition typically lasted well over 1 min in the majority of cases, with a trough up to 19 mV below resting membrane potential (RMP; Fig. 4*A*,*B*). Recorded neurons were routinely filled with neurobiotin so their anatomy (Fig. 4*C–E*) and its relation to the KO inhibition could be examined. There was a correlation between peak conductance increases during KO and the longitudinal location of neurons (*n* = 48 dINs and 25 non-dINs analyzed; Fig. 4*F*). Neurons less than ∼750 µm from the mid/hindbrain border tended to have clear increases in the membrane conductance. Peak conductance increases were also correlated with the amplitudes of KO inhibition (Fig. 4*G*), confirming that KO inhibition was mediated by the opening of some ion channels. The half-trough duration of KO inhibition in dINs was longer at more rostral positions (*n* = 26, *p* < 0.01, correlation coefficient 0.52). Although most neurons were recorded opposite to the stimulated side for access reasons, neurons on the stimulated side also received KO inhibition (*n* = 3). Increasing the number of stimulation pulses could induce larger rises in the peak conductance during KO inhibition (*p* < 0.05, *n = 10* dINs, related samples Wilcoxon signed rank test; Fig. 4*H*). This is in line with the observation in physically restrained tadpoles that multiple taps stop swimming more quickly.

**Figure 5.**
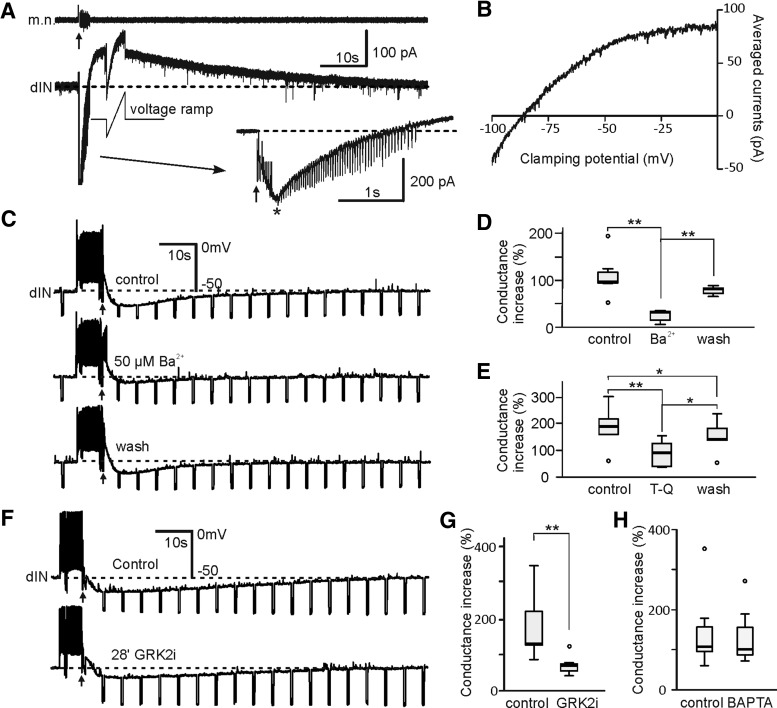
KO inhibition induced by electrical head skin stimulation (arrows) is mediated by GIRKs. ***A***, A voltage-clamp recording shows the slow outward KO current and its onset (inset, *) after KO stimulation (five pulses at 30 Hz). A voltage ramp rising from −100 to 0 mV is applied at the peak of the current. Membrane potential is held at −60 mV at rest. ***B***, I-V data points averaged from five recordings during the voltage ramp in ***A*** for estimating the reversal of the currents and their rectification. ***C***, Effects of microperfusing 50 µM Ba^2+^ on KO inhibition in a dIN. ***D***, Summary of blockade of peak conductance rises by 50–100 µM Ba^2+^ during KO in two dINs and six non-dINs (paired *t* tests). ***E***, Microperfusing 3 µM Tertiapin-Q (T-Q) weakened the peak conductance increase during KO in one dIN and five non-dINs (paired *t* tests). ***F***, One dIN recording with a pipette solution containing 10 µM GRK2i at the beginning (control) and after 28 min of equilibration. ***G***, GRK2i weakened the peak conductance increase during KO inhibition (*n* = 8, paired *t* test). ***H***, Summary of peak membrane conductance increase during KO in control and after BAPTA equilibration (*n* = 8, related samples Wilcoxon signed rank test, *p* = 0.33). *, significance at *p* < 0.05 and ** at *p* < 0.01 in ***D***, ***E***, ***G***. Dashed lines indicate RMP or clamping current at −60 mV in ***A***, ***C***, ***F***.

To identify which type of ion channel mediated the KO inhibition, we recorded neuronal responses in voltage-clamp mode. Slow outward currents were recorded coinciding with KO responses in the motor nerve recordings (Fig. 5*A*). Estimated by the turning point of the current trajectory, the average onset time for these outward currents from KO stimulation was 343 ± 56 ms (*n* = 6 dINs; Fig. 5*A*, inset). Ramp voltage tests applied at the peak of the outward currents revealed a reversal of –88 ± 4.9 mV, with inward rectification at −52.8 ± 3.4 mV (*n* = 6 dINs; Fig. 5*B*). Ba^2+^ at 50 µM in one dIN and 100 µM in another dIN and six non-dINs, microperfused close to the recorded neuron, could reversibly block the peak membrane conductance increase by 77.9 ± 3.2% (Fig. 5*C*,*D*). Microperfusing 3 µM Tertiapin-Q, a blocker for inward-rectifier K^+^ channels, reduced peak conductance increase by 50.7 ± 7% (*n* = 1 dIN and *n* = 5 non-dINs; Fig. 5*E*). However, tertiapin-Q can affect Ca^2+^-dependent potassium currents (Kanjhan et al., 2005). Therefore, we recorded neurons with electrodes containing 20 mM BAPTA to chelate free cytoplasmic Ca^2+^ to block Ca^2+^-dependent processes like the opening of Ca^2+^-dependent potassium currents. This was done in 3 sequential recordings where neurons were first recorded with electrodes containing normal pipette solution and five other recordings just with BAPTA electrodes. Comparing membrane conductance increase during KO inhibition in control with that after 15–60 min of BAPTA equilibration revealed no difference (*n* = 2 dINs and *n* = 6 non-dINs in eight tadpoles; Fig. 5*H*), thus excluding the involvement of Ca^2+^-dependent potassium currents in the KO inhibition. We next included 10 µM GRK2i in the pipette solution to inhibit G protein βγ-subunit in five dINs and three non-dINs. GRK2i reduced the peak conductance rise during KO inhibition by 57.6 ± 4.2% (Fig. 5*F*,*G*). The reduction was not correlated with GRK2i equilibration time (two-tailed Pearson, *p* = 0.27), suggesting part of the conductance rise results from the opening of K^+^ channels in neighbouring, electrically coupled neurons (Li et al., 2009). These current properties and the pharmacological tests support that G protein-coupled inward-rectifying potassium channels (GIRKs; Dutar et al., 1999; Lüscher and Slesinger, 2010) mediate the KO inhibition.

**Figure 6.**
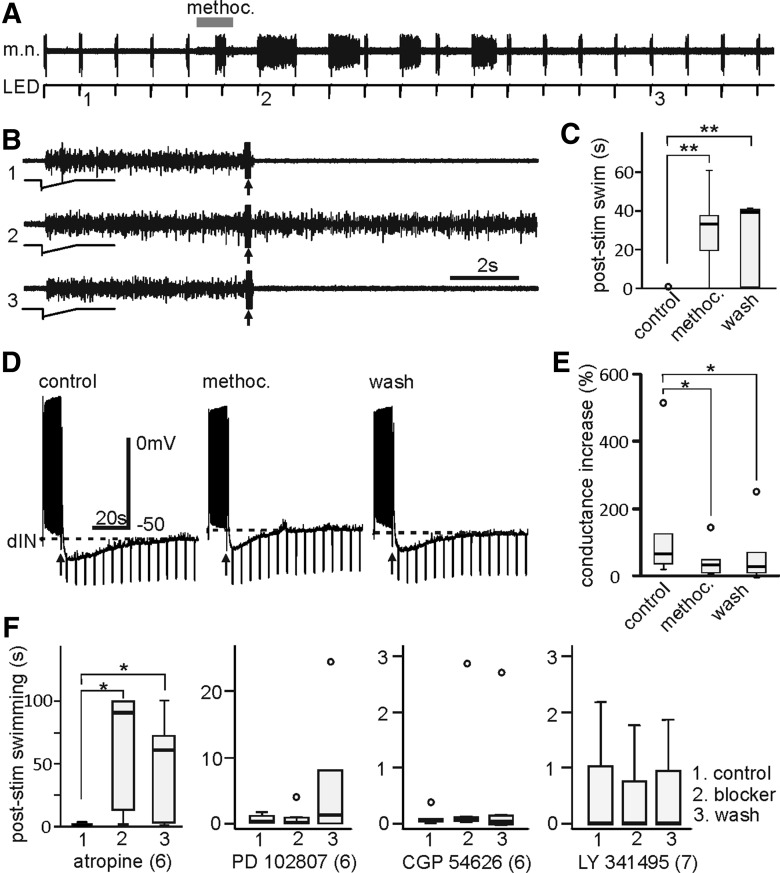
The pharmacological blockade of KO responses and KO inhibition. ***A***, KO responses induced by KO stimulation (arrow, five pulses at 30 Hz) applied every 100 s. Swimming (m.n.) is evoked by dimming an LED. Gray bar indicates time of the application of 10 µM methoctramine (methoc., 100 s). ***B***, Example KO responses before (1), shortly after methoc. application (2), and recovery (3). ***C***, KO responses are blocked 200 s after methoc. application (*p* < 0.01, *n* = 9 tadpoles, related samples Wilcoxon signed rank test) but without recovery 25 min after wash. ***D***, KO inhibition following skin stimulation (arrows, five at 30Hz) in a dIN is reduced by the microperfused 10 µM methoc. Swimming is initiated by dimming an LED light. ***E***, Conductance increases during KO inhibition are weakened by methoc. (*n* = 6, *p* < 0.05, related samples Wilcoxon signed rank test). ***F***, Summary of post-KO swimming lengths measured at 0, 200, and 2500 s from the application of different antagonists (numerals in brackets indicate number of tadpoles tested, related samples Wilcoxon signed rank tests). Atropine (10–50 µM) is a nonselective muscarinic receptor blocker. PD102807 (0.5 µM) is a selective M_4_ muscarinic receptor blocker. CGP54626 (10 µM) is a GABA_B_ receptor blocker and LY341495 (10 nM) blocks mGluR II and III. *p < 0.05 and **p < 0.01 in ***C***, ***E***, ***F***.

We next determined what type of transmitter receptor is coupled to the GIRKs to initiate KO inhibition. Activation of postsynaptic receptors, like GABA_B_ (Newberry and Nicoll, 1985), mGluRII (Dutar et al., 1999), and M_2_ muscarinic receptors (McCormick and Prince, 1986) can all open GIRKs. In tadpoles, GABAergic, glutamatergic and cholinergic transmissions have been reported (Roberts et al., 2010). We initially microperfused the nonselective muscarinic receptor antagonist, atropine (10–50 µM), to the hindbrain in six tadpoles. Atropine weakened KO responses (*p* < 0.05, related sample Wilcoxon signed rank test) without recovery during wash (Fig. 6*F*). Bath application of 10 µM methoctramine, an M_2/4_ receptor blocker, reliably blocked the KO responses, allowing swimming after KO stimulation to carry on for 33.5 s (range: 0.34–61.1, *n* = 9 tadpoles, *p* < 0.01; Fig. 6*A—C*). In accord with this, microperfused methoctramine (10 µM) also weakened the KO inhibition in the recorded neurons by 55 ± 6.9% (Fig. 6*D*,*E*). A selective M_4_ receptor antagonist, PD102807 at 0.5 µM, did not affect KO responses (Fig. 6*F*). Bath application of a GABA_B_R antagonist, CGP 54626 at 10 µM and a mGluR II and III (Chapman and Sillar, 2007) antagonist, LY341495 at 10 nM did not have any effect on KO responses (Fig. 6*F*). These data support that stimulating the head skin activates some cholinergic neurons and that GIRKs are coupled to postsynaptic M_2_ muscarinic receptors to mediate KO inhibition.

**Figure 7.**
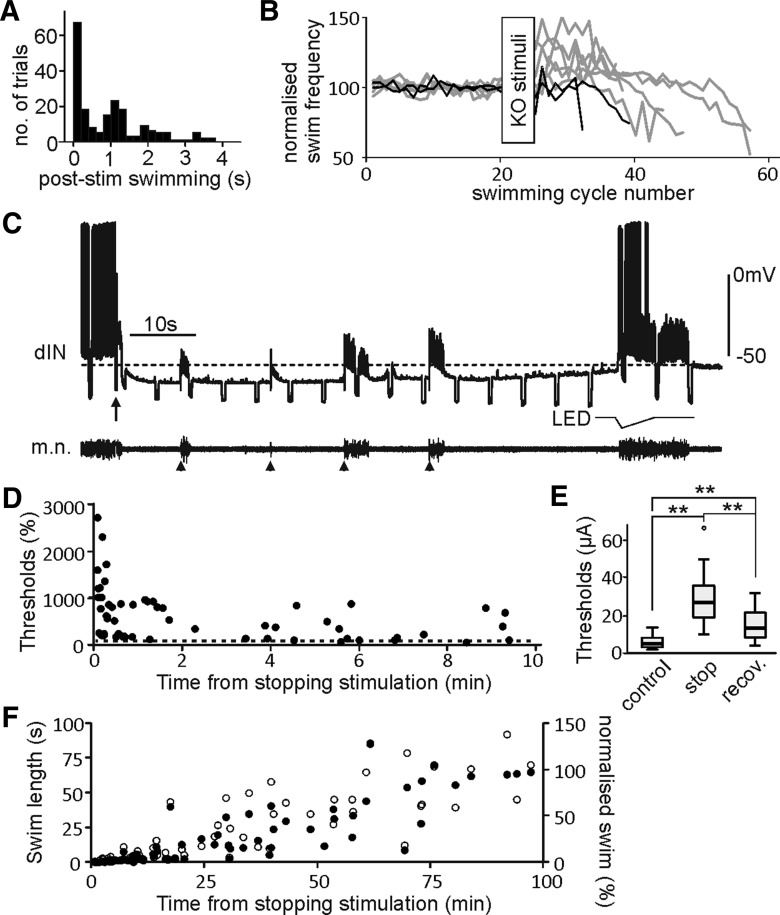
Swimming activity after KO stimulation. ***A***, Distribution of swimming lengths after KO stimulation (range: 0–3.81 s, *n* = 197 trials). Trials are chosen from 20 tadpoles where membrane conductance during KO has increased by >100% (50/197 without swimming preceding KO). ***B***, Normalized swimming frequency before and after KO stimuli. Gray traces are six trials with frequency increases (*p* < 0.05, *t* test, 10 post-KO swimming cycles compared with control). ***C***, Motor responses following KO stimulation (arrow). Four single stimuli (arrow heads, 220 µA) are applied to evoke swimming during KO inhibition. Note failures in dIN spiking. ***D***, Swimming thresholds (normalized to control, dashed line) tested with single skin stimulation after KO. ***E***, Swimming thresholds are higher after KO stimulation and their recovery >5 min later (*n* = 10 sites in six tadpoles, *p* < 0.001, related samples Friedman’s two-way ANOVA by ranks). ***F***, Lengths of swimming evoked by single suprathreshold skin stimulation after KO stimulation (filled circles). Empty circles show the same normalized data (secondary axis).

A common feature of concussion is the loss of motor control and we asked how tadpole swimming was affected by KO. Immediately after KO stimulation, swimming could stop straight away or carry on for seconds depending on the location and intensity of stimulation (Fig. 7*A*; also see Figs. 3*A*,*C*,*G*; 4*A*,*B*,*H*; 5*A*,*C*,*F*; 6*B*,*D*). We analyzed trials where swimming did not stop immediately, and also whole-cell recordings had revealed >100% conductance increases during KO inhibition. There was normally a transient increase followed by a rapid decrease in swimming frequencies before the end of swimming (Fig. 7*B*). We have previously shown dINs are critical for swimming initiation and maintenance (Roberts et al., 2010; Moult et al., 2013). In agreement with the inhibition of rostral dINs by KO stimulation (Fig. 4*F*), swimming initiation thresholds for electrical stimulation increased for >10 min after KO stimulation (Fig. 7*D*,*E*). When swimming was induced by suprathreshold stimulation, swimming lengths were shortened for ∼1 min after KO stimulation (Fig. 7*C*,*F*). These results show tadpole motor responses are significantly suppressed immediately after KO stimulation.

**Figure 8.**
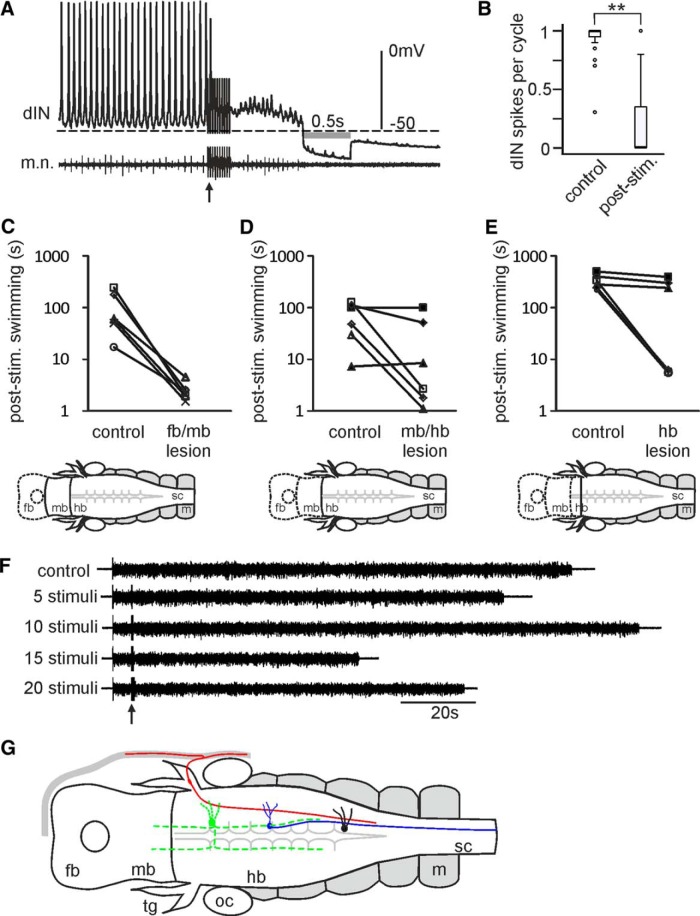
Locating neurons responsible for KO responses. ***A***, A dIN recording after KO stimulation (arrow, 10 pulses at 30 Hz). Gray bar marks the R_inp_-testing period. ***B***, dIN firing reliability decreases after KO stimulation (**p < 0.001, *n* = 19 trials from 10 tadpoles, related sample Wilcoxon signed rank test). ***C***, KO responses persist with lesions at forebrain and midbrain border (fb/mb). ***D***, KO responses persist in three out of six tadpoles with lesions at the midbrain and hindbrain border (mb/hb). ***E***, KO responses persist in three out of six tadpoles with lesions at the rostral edge of trigeminal nerve entry to hindbrain. Diagrams below bar charts in ***C****–****E*** show lesion locations (dashed lines outline removed brain). Each line connects averaged swimming lengths from more than five trials in control and with KO stimuli (5 at 30 Hz and 320 µA). Empty symbols show tadpoles where KO persists after lesions and filled symbols represent tadpoles with KO responses abolished by the lesion (independent samples *t* test or median test, *p* < 0.05 in each tadpole). ***F***, Swimming of a tadpole with KO responses abolished by the lesion at the rostral edge of the trigeminal nerve entry to the hindbrain. Arrow indicates time of electrical skin stimuli. Swimming is initiated by a single electrical stimulus to the head skin. ***G***, Diagrammatic summary of the KO pathway (dorsal view) mediating the loss of motor responses. Stimulating the head skin excites the peripherals of rapid-transient detectors (red) located in the trigeminal ganglion, which in turn activate the unidentified cholinergic interneurons (green with dashed processes) in the brainstem. The cholinergic cells inhibit the rostral dINs (blue) by opening the GIRKs coupled to M_2_ muscarinic cholinergic receptors. The non-dINs (black) then lose the excitatory inputs from dINs and tadpole motor responses are suppressed. For abbreviations, see Figure 3*E*.

We next tried to identify where the cholinergic interneurons responsible for KO responses are located. dINs in the tadpole swimming circuit corelease glutamate and ACh, activating postsynaptic AMPA, nicotinic acetylcholine and NMDA receptors (Li et al., 2004b). The coreleased ACh could theoretically also activate M_2_ receptors and mediate KO. However, there was a decrease in dIN spiking after the KO stimulation in comparison to its reliable firing in control (Fig. 8*A*,*B*). Therefore, dINs in the hindbrain are unlikely to be the source of KO inhibition. Several groups of cholinergic neurons have been identified in tadpole mid- and hindbrain (López et al., 2002). We made transections at three locations of the tadpole CNS to locate the KO-mediating cholinergic neurons: the forebrain and midbrain border, the mid- and hindbrain border and the rostral edge of the trigeminal nerve entry point to the hindbrain (Fig. 8*C–E*). More caudal lesions were not possible since the trigeminal nerves are required to induce KO. Removing the forebrain did not abolish KO responses in six out of six tadpoles. KO responses disappeared in three out of six tadpoles in the other two types of more caudal lesions. Figure 7*F* shows the failure to evoke KO responses even when the number of KO stimuli was increased to 20 and at maximum current intensity of 320 µA. This suggests midbrain and hindbrain areas are important for the KO responses.

## Discussion

In this study, we have revealed a novel cellular mechanism that can mediate a behavior resembling physical KOs, a mild form of concussion observed in most vertebrates. We find mechanosensory stimulation to the head skin can reliably evoke KO, contrary to the prevalent view that concussion is caused by direct physical impacts to the brain. The tadpole head skin is innervated by two types of sensory cells located in the trigeminal ganglia: rapid-transient detectors and slow movement detectors (Roberts, 1980; Hayes and Roberts, 1983) with distinctive peripheral neurites (Roberts and Blight, 1975). Our results show only the activation of rapid-transient detectors evokes KO inhibition (Fig. 2), in agreement with the observation that concussive blows have to reach certain speed to be effective (Shaw, 2002).

How does the tadpole KO behavior compare with concussion in other animal models? Although the general criteria for the definition and diagnosis of concussion have not been agreed on among medical professionals (Blennow et al., 2012) and proposed concussion mechanisms vary with animal models employed, the methods used to induce concussion and the severity of concussion, there are some common observations.

Immediately after concussive injuries, there is an increase in ACh in the cerebrospinal fluid in many experimental animals (Bornstein, 1946; Tower and Mc, 1948; Sachs, 1957) and patients (Tower and Mc, 1948; Sachs, 1957). The involvement of cholinergic neuronal activity has been the foundation of the pontine cholinergic hypothesis, where an ACh increase has been attributed to elevated cholinergic activity in the dorsal pontine tegmentum in mammals (Saija et al., 1988). In line with the importance of cholinergic transmission in the concussion response, muscarinic antagonists like atropine or scopolamine have been shown to provide some beneficial properties in comatose head injury patients and experimental animals (Bornstein, 1946; Ward, 1950; Ruge, 1954; Sachs, 1957; Lyeth et al., 1988). Microinjection of carbachol into the pontine tegmentum region can also induce behavioral symptoms of concussion, including loss of muscle tone, flexion, righting and placing reflexes (Ommaya and Gennarelli, 1974; Hayes et al., 1984). However, the pontine cholinergic system has now been well established as critical in inducing a REM sleep-like state (Shiromani et al., 1987; Jones, 1991). Without a cellular level of understanding of exactly what role ACh plays in concussion, it is not clear whether the rise in ACh levels, like the rise in glutamate and aspartate (Nilsson et al., 1990), is the cause of concussion or a by-product of concussion (Nilsson et al., 1994). There is also doubt that animals in the carbachol-induced concussion-like state are awake (George et al., 1964; Mitler and Dement, 1974; Velasco et al., 1981) because some reflexes persist. Our data clearly show that cholinergic inhibition is directly responsible for the depression of motor responses following KO stimulation and also reveal that the inhibition is mediated by the opening of GIRKs.

The question then is whether the cholinergic inhibition comes from the pontine region in tadpoles. Eight groups of cholinergic neurons, including some motoneurons, have been identified by choline acetyltransferase immunoreactivity in the mid- and hindbrain of stage 39/40 *Xenopus* tadpoles (López et al., 2002). Our lesion experiments (Fig. 8) show that removing the midbrain and the first hindbrain rhombomere segment, which contain the equivalent pontine region in higher vertebrates, abolishes KO responses only in some tadpoles. This implies that some KO-mediating cholinergic interneurons are located in the more caudal hindbrain, whereas others are rostral to the entry point of trigeminal nerves. Since trigeminal axons all descend caudally toward and into the spinal cord (Hayes and Roberts, 1983), there must be some sensory interneurons with ascending axons relaying the trigeminal mechanosensory information to these cholinergic interneurons. In the meantime, either the sensory interneurons or the cholinergic interneurons should possess commissural axons because neurons on both sides receive KO inhibition. The muscarinic inhibition during KO is surprising because muscarinic control of locomotion by the mesencephalic locomotor region (MLR; which include the cholinergic pedunculopontine nucleus) is generally excitatory (Sholomenko et al., 1991; Sirota et al., 2000; Smetana et al., 2007). For example, the MLR in lampreys excites muscarinoceptive neurons in the rostral brainstem by activating muscarinic receptors, which in turn sends glutamatergic excitation to reticulospinal neurons to boost locomotion in a feed forward manner (Smetana et al., 2010). Further work is needed to fully delineate the neural pathways involved in the KO responses.

Another common observation in many concussion models is the marked rise of extracellular potassium concentration ([K^+^]_o_) in the cerebral cortex, brainstem (Takahashi et al., 1981), and hippocampus (Katayama et al., 1990), presumably as a consequence of mechanically induced excessive, epileptic-like neural activity from activating mechanosensitive ion channels (Morris, 1990; Sachs, 1991). The opening of K^+^ currents can provide a straightforward explanation for such [K^+^]_o_ rise. Our data support the K^+^ currents involved in tadpole KO responses are mediated by GIRK channels. The fast onset of KO inhibition (∼343 ms) can explain why most KO responses take place instantaneously after the concussive blow, although in tadpoles swimming can still carry on for a few cycles because its frequency is between 10–25 Hz. Apart from its fast onset, our data show the KO inhibition is sensitive to extracellular Ba^2+^, tertiapin-Q and intracellular GRK2i application. Ba^2+^ at concentrations below 200 µM is more specific for K^+^ currents with inward rectifying properties (Chen and Johnston, 2005), but at higher concentration, it also blocks A-type K^+^ channels (Gasparini et al., 2007). Our previous study, however, failed to identify A-type K^+^ channels in dINs (Li, 2015). The more specific GIRK channel blocker, Tertiapin-Q (Jin et al., 1999), also blocks Ca^2+^-activated large conductance K^+^ channel (Kanjhan et al., 2005). The involvement of Ca^2+^-dependent K^+^ channels in KO inhibition is unlikely since BAPTA electrodes do not change the rise in conductance during KO inhibition (Fig. 5*H*). This fits in with decreased dIN activity after KO stimulation, which is unlikely to lead to increased Ca^2+^-dependent K^+^ conductance. Indeed the weakening of KO inhibition by intracellular GRK2i provides more direct evidence that GIRKs mediate KO inhibition. The rise in [K^+^]_o_ is unlikely the result of mechanical stimulation of neurons because electrical head skin stimulation can reliably evoke KO inhibition. Previously, during dual electrode recordings from the same neurons (Li et al., 2004a), the monitoring electrode has never recorded any increase in neuronal activity due to the mechanical stress caused by the second electrode. In contrast, spiking in many neurons is often reduced or stopped by KO stimulation in tadpole KO responses (Fig. 8). Therefore, it is unlikely that the [K^+^]_o_ increase is a result of excessive neuronal activity.

Testing KO inhibition against different receptor antagonists suggest that GIRKs are coupled to muscarinic receptors (Fig. 6). The M_2_ and M_4_ subtypes of muscarinic receptors are known to mediate inhibitory neuronal responses. Unfortunately, available M_2_ antagonists all have similar affinity to M_4_ receptors. Tadpole KO responses are sensitive to methoctramine but not to PD102807, a more selective M_4_ receptor blocker. Therefore, independent means to test the viability of PD102807 in tadpoles is lacking. Published data, most notably from the sympathetic innervation of heart cells, show GIRKs are coupled to M_2_ muscarinic receptors, giving GIRKs the name of muscarinic potassium channel (Wickman et al., 1999). Although more definitive identification of muscarinic receptors here still awaits antagonists with better subtype specificity, existing data support that these GIRKs are likely coupled to M_2_ receptors.

Finally, in terms of animal behavior, concussion is defined by the consistent loss of motor responses after the concussive blow. The convulsive hypothesis has attributed this loss of motor function to the excessive neuronal activity immediately following the concussive blow. In tadpole KO responses, the onset of full GIRK inhibition is slow, allowing swimming activity sometimes to carry on for several seconds after the KO stimulation (Fig. 7*A*). During that period, the normal mechanosensory inputs can provide extra excitation to the swimming circuit (Buhl et al., 2012) and temporarily increase swimming frequency (Fig. 7*B*). This might be equivalent to the convulsive motor activity often observed immediately following the concussive blow in other concussion models. While other competing hypotheses have failed to provide a clear cellular mechanism to explain the loss of motor control during concussion, the convulsive theory proposes that “spreading depression” (Sugaya et al., 1975) following the convulsive, initial excitation phase may exert the wide-spread suppression of brain functions. One proposed cause of the depression is the activation of Ca^2+^-dependent potassium channels following Ca^2+^ influx during the convulsive excitation phase, similar to what occurs in the case of epileptic bursting (Hotson and Prince, 1980). Our data show that KO inhibition is not sensitive to the chelating of intracellular Ca^2+^. This is in line with the observation that KO inhibition can be evoked in many cases without any preceding swimming activity. A second proposed cause for the spreading depression is the increase in ion pump activity (Mayevsky and Chance, 1974) after the convulsive neuronal activity. This is thought to be the main cause for subsequently increased glucose consumption, depletion of energy stores and acute failure in neuronal functions (Giza and Hovda, 2001; Shaw, 2002; Blennow et al., 2012). Increased Na^+^/K^+^ pump activity can quickly hyperpolarise membrane potentials for many seconds (Parker et al., 1996; Pulver and Griffith, 2010; Zhang and Sillar, 2012). However, Na^+^/K^+^ pump activity is dependent on preceding neuronal spiking and its inhibition of membrane potentials does not change the cellular conductance. Therefore, it is unlikely for increased Na^+^/K^+^ pump activity to mediate the tadpole KO inhibition. Instead, the GIRK-mediated inhibition directly suppresses the excitability of dINs located in the hindbrain, markedly increasing swimming initiation thresholds and shortening swimming episode length (Fig. 7). This fits in well with the critical roles of dINs in swimming rhythmogenesis (Roberts et al., 2010; Moult et al., 2013).

Apart from the loss of motor control, concussion in mammals and primates is often accompanied by the loss of cerebral consciousness (Leclerc et al., 2001; Shaw, 2002). At this stage, it is thus difficult for us to fully relate the tadpole KO response to concussion in higher vertebrates because the tadpole forebrain is still largely undeveloped. Nevertheless, adult frogs have previously been used as a concussion model (Denny-Brown and Russell, 1941). It would be interesting to study whether similar KO inhibition is responsible for the concussion in older tadpoles/adult frogs. We also need to identify the KO-mediating cholinergic neurons and their projection patterns so we can evaluate more precisely which brain functions are potentially affected by concussion. Undoubtedly, direct damage to the brain tissue can mediate longer-term concussion symptoms, especially in case of repeated concussion. The tadpole KO shares a high similarity in behavior, the depression of motor responses, the duration of motor inhibition and the induction sites with concussion in other animal models. Here, we propose the muscarinic receptor-coupled GIRK-mediated inhibition is potentially an evolutionarily conserved mechanism that can mediate some acute symptoms of concussion in vertebrates.

## Synthesis

Reviewing Editor: Michael Fainzilber, Weizmann Institute of Science Decisions are customarily a result of the Reviewing Editor and the peer reviewers coming together and discussing their recommendations until a consensus is reached. When revisions are invited, a fact-based synthesis statement explaining their decision and outlining what is needed to prepare a revision will be listed below. The following reviewer(s) agreed to reveal their identity: Carlos Aizenman, Elizabeth Coulson.

The manuscript is an interesting study regarding potential mechanisms that might explain some features of concussion, which are not due to brain damage.

While a tadpole's head bumping into a surface (in nature, a log or rock) may be quite different to that of a mammal crashing into a window or wall, the authors propose an interesting idea (albeit requiring significant future testing to verify) from their results; The main thrust of the paper appears that the contact of the head/skin with the object modulates trigeminal neurons via cholinergic M2 receptor activation of GIRK channels, resulting in inhibition of swimming action of the tadpoles.

The manuscript is interesting and most of the previous comments were addressed. The involvement of GIRK channels is as robust as pharmacology allows. However, some aspects of the study are still confusing, particularly the paradigms and the proposed circuitry, as detailed below:

1. The abstract should mention in which cells the GIRKs are active.

2. In the introduction, the authors state when other animals crash..“their movement often stops abruptly” The extrapolation of what is being studied in the tadpole is a little unclear - it seems the experimental paradigm is that the tadpole is hit/stimulated several times before it is rendered immobile ~1 second later ie after 5 head bumps? (pg 8). Does this mean there is a threshold/additive effect, and/or suggest there is quite a different mechanism in play to what occurs in concussion? This requires clarification and consistency with what is said in the introduction about the tadpoles and the methods used for the frog-mammal extrapolation to be considered valid.

3. In the methods it is unclear where the vision-guided whole-cell recordings were taking place - the trigeminal ganglion? Does this ganglion contain both dIN and non dIN neurons? Are all the recordings from cells in this ganglion? see point 10 below.

4. Pg 7 line 164 something is missing from the methods?

5. It was not clear when stimulation parameters varied per animal why that was. Were you aiming to get a consistent output from each animal to then explore mechanisms from a consistent baseline, or were you trying to understand the parameters that caused an output, or was it unplanned variability, or other. This needs to be clarified (perhaps in the methods, and removed from the results if not relevant).

6. Pg 9 line 249 - Whole-cell recordings showed KO inhibition was always present if neurons in the rostral hindbrain were recorded simultaneously. Is this suggesting that the simultaneous recoding somehow influences the KO response, or a statement that inhibition occurred in all those experiments when there happened to be simultaneous recording (which is a methodological fact, not a result).

7. Fig 3I. its not clear that black circles in the figure corresponds to a KO response whereas white indicates no knockout - has this been quantified or is the 60% cited in text an estimate? It seems odd that tail level stimulation would produce a concussion response. Also that many head stimulations did not. This calls into question the extrapolation that the stimulation is mimicking a concussion event rather than a reflex to stop movement in a physically blocked direction. When I walk into a chair and hurt my knee I stop travelling (in that direction) and pause, but I am not concussed.

8. In Fig 4 it is unclear to me where in the tadpole the (non-) dIN cells being recorded from are. There are many more black cells in the scatter graphs 4 F and G than are on the Fig H. what does that signify? I do not understand what the “5/20” numbers are in H “H. Increasing the number of electrical skin stimuli (paired numerals above bars) while maintaining the stimulation strengths and frequencies induces”. is it number of stimulations (5) /Hz (20)?

9. Fig 5E pg 13/14 It is counter intuitive that stimulation of both types of cell (non dIN vs dIN) at the head or tail makes little difference to the output? How are these cell types different ? I presumed their input and output circuitry was different? but then they shouldn't be interchangable in this experiment. Clarify in text. Perhaps a wiring diagram or better introduction would clarify.

10. Slow outward currents were recorded coinciding with KO responses ***in the motor nerve recordings**** (Fig.5A). How does this recording site relate to the trigeminal recordings in pt 3 above? Is there more than one recording site? Is the cell body or a axonal/dendritic response being recorded?

11. Related, if there is a motor neuron component ( which is therefore cholinergic/ muscarinic), the effects are perhaps not surprising (block a MN, animal stops moving) - and calls into question whether the response is a reflex in response to touch, or truly an aspect to concussion (see point 7). If the former, the justification for the study (seeking to understand concussion specifically) requires modification but is still of interest.

12. Line 310 “This was done in 3 sequential recordings where neurons were first recorded with electrodes containing normal pipette solution and 5 other recordings just with BAPTA electrodes.” The wording is odd - 3 + 5 sites per animal? Was the internal solution exchanged or new recording electrodes used? Was the recording paradigm merely repeated 5 times? Is it not atypical for many iterations of a recording sequence to be performed on each patched cell ? Is this something different?

13. Pg 14 line 315. While tertiapin can affect Ca-sensitive K channels (as cited), BAPA would affect function of all Ca dependent channels (and other dependent processes) not just K channels. This should be clarified.

14. Bath application of methoctramine studies are confusing. “allowing swimming after KO stimulation to carry on for 33.5 s (range: 0.34 - 61.1, n = 9 tadpoles, p < 0.01, Fig.6A-C).” Does this mean swimming could carry on for up to 33.5 second? On average? did it then stop? why? Furthermore, if the experiment is inhibiting motor neurons ( by use of cholinergic antagonists) is it assumed you would stop motor function i.e. you will stop swimming... therefore is this a circular argument? see point 11.

15. Fig 7. C,F I don't understand the relevancy of these data - is the conclusion that the effect is to make it harder to initiate swim after KO? explain.

16. The final sentence of the results is not accurate. “This suggests that the cholinergic interneurons are longitudinally distributed in the midbrain and hindbrain.” This is an anatomical statement that is presumably testable by other means, which is not what the study has undertaken. Rather, are the authors saying that midbrain and hindbrain areas are important for their response? - Note that presumably such neurons could the triggering cholinergic neurons elsewhere? ie indirectly not necessarily directly

17. Fig 1 B presents only ~8 trials where multiple head strokes were tested (in black)? Is this data relevant? it may be simpler to remove it. What is the point of including such data given the range of unstimulated responses of the tadpoles.

18. The authors should display their data as box plots or showing individual data points as a cluster plot rather than a mix of graph styles. It is very hard to determine the real spread of the data from the bar graphs, especially when differences are small.
